# Supplementary material for: Complex‐centric proteome profiling by SEC‐SWATH‐MS
Source: Mol Syst Biol. 2019 Jan 14;15(1):e8438. doi: 10.15252/msb.20188438 (PMC6346213; doi:10.15252/msb.20188438)
Supplement: Supplementary file 8 — Dataset EV7 [file MSB-15-e8438-s008.zip › feature_plots_string/P36507.pdf]

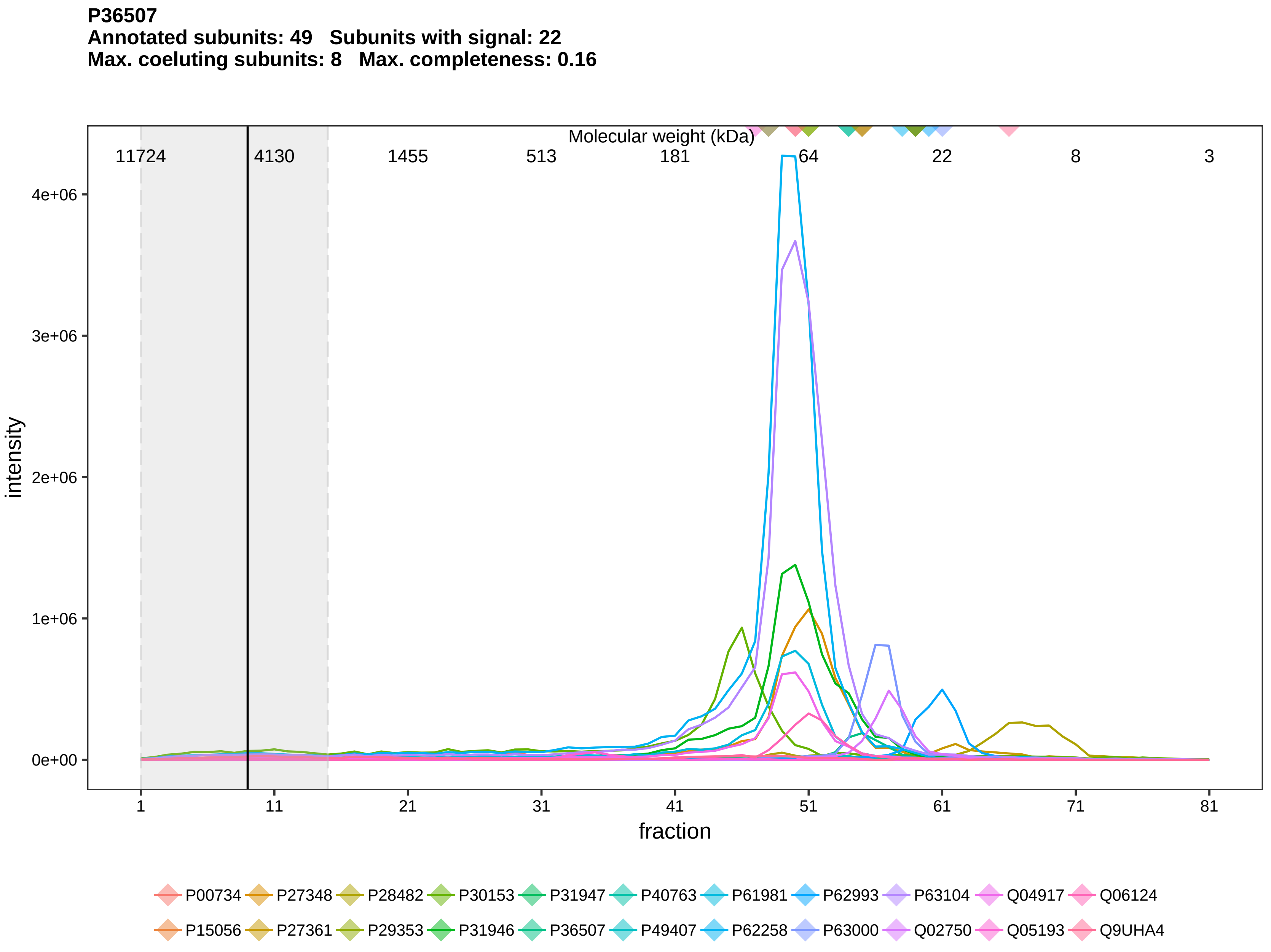
P36507
Annotated subunits: 49   Subunits with signal: 22
Max. coeluting subunits: 8   Max. completeness: 0.16
Molecular weight (kDa)
11724
4130
1455
513
181
64
22
8
3
intensity
4e+06
3e+06
2e+06
1e+06
0e+00
fraction
1
11
21
31
41
51
61
71
81
P00734
P27348
P28482
P30153
P31947
P40763
P61981
P62993
P63104
Q04917
Q06124
P15056
P27361
P29353
P31946
P36507
P49407
P62258
P63000
Q02750
Q05193
Q9UHA4
